# Dendritic Polyglycerol Amine: An Enhanced Substrate to Support Long-Term Neural Cell Culture

**DOI:** 10.1177/17590914211073276

**Published:** 2022-01-13

**Authors:** Jean-Pierre Clément, Laila Al-Alwan, Stephen D. Glasgow, Avya Stolow, Yi Ding, Thaiany Quevedo Melo, Anouar Khayachi, Yumin Liu, Markus Hellmund, Rainer Haag, Austen J Milnerwood, Peter Grütter, Timothy E. Kennedy

**Affiliations:** 1 Program in Neuroengineering, Montreal Neurological Institute, 98613 McGill University, Montreal, Canada; 2 Department of Neurology and Neurosurgery, Montreal Neurological Institute and Hospital, Montreal, Canada; 3 Department of Physics, 12367 McGill University, Montreal, Canada; 4 Institute of Chemistry and Biochemistry, 197690 Freie Universität Berlin, Berlin, Germany

**Keywords:** cell survival, cell differentiation, dendritic polyglycerol amine, human iPSCs, neural culture, surface coating

## Abstract

Long-term stable cell culture is a critical tool to better understand cell function. Most adherent cell culture models require a polymer substrate coating of poly-lysine or poly-ornithine for the cells to adhere and survive. However, polypeptide-based substrates are degraded by proteolysis and it remains a challenge to maintain healthy cell cultures for extended periods of time. Here, we report the development of an enhanced cell culture substrate based on a coating of dendritic polyglycerol amine (dPGA), a non-protein macromolecular biomimetic of poly-lysine, to promote the adhesion and survival of neurons in cell culture. We show that this new polymer coating provides enhanced survival, differentiation and long-term stability for cultures of primary neurons or neurons derived from human induced pluripotent stem cells (hiPSCs). Atomic force microscopy analysis provides evidence that greater nanoscale roughness contributes to the enhanced capacity of dPGA-coated surfaces to support cells in culture. We conclude that dPGA is a cytocompatible, functionally superior, easy to use, low cost and highly stable alternative to poly-cationic polymer cell culture substrate coatings such as poly-lysine and poly-ornithine.

Summary statement

Here, we describe a novel dendritic polyglycerol amine-based substrate coating, demonstrating superior performance compared to current polymer coatings for long-term culture of primary neurons and neurons derived from induced pluripotent stem cells.

## Introduction

Neurons are adherent cells that require an appropriate substrate to survive, differentiate, and elaborate axons and dendrites (Banker & Goslin, 1998). In fact, the critical significance of providing an appropriate substrate was identified in the earliest cell culture studies, supporting the conclusion that process extension by adherent cells requires a solid substrate (Harrison, 1914). More contemporary *in vitro* studies have revealed that depriving adherent cells of a supportive matrix initiates Anoikis, a form of apoptotic cell death triggered by loss of adhesion (Frisch & Francis, 1994; Meredith et al., 1993). While some cells, including many immortalized cell lines, can attach and grow on bare borosilicate glass (Stoker et al., 1968) or tissue culture-treated polystyrene, which is usually oxidized via plasma treatment to increase its hydrophilicity (Lerman et al., 2018), many primary cells, including most neuronal cells, do not sufficiently adhere or survive on the plastic or glass surfaces of cell culture vessels. To adhere and survive, these cells require a more engaging substrate that mimics at least some of the properties of a physiological extracellular matrix (ECM).

Various ECM components have been employed for this purpose, including collagen, fibronectin, laminins, and ECM extracts like Matrigel™ to support the attachment and proliferation of neuronal cells (Kleinman et al., 1981, 1987), however, these ECM components are challenging to purify, expensive to purchase and prone to variation in quality. To address these issues, synthetic poly-peptides were introduced as a relatively inexpensive alternative to natural ECM components to coat cell culture substrates (Letourneau, 1975; McKeehan & Ham, 1976; Yavin & Yavin, 1974). Poly-lysine and poly-ornithine are homopolymeric chains of a basic amino acid that were selected based on early observations that proteins with a high content of positively charged amino acids, such as histones and protamine, when adsorbed onto a cell culture substrate would support cell adhesion and proliferation (McKeehan & Ham, 1976). More recent studies have reported that non-peptide polymers such as polyethyleneimine (Vancha et al., 2004), polypropyleneimine (Lakard et al., 2005), polypyrrole (Schmidt et al., 1997), poly(allylguanidine) (Ji et al., 2019) and poly-electrolyte multilayers (PEMs) (Landry et al., 2018) can function as substrates to support neural cell culture. The unifying characteristic of these polymers is that they all contain basic amine functional groups, suggesting that a relatively high density of positive charge at physiological pH is the key parameter to promote cell adherence to the surface.

Poly-lysine and poly-ornithine are commonly used as substrate coatings for primary cells and cell lines in contemporary cell culture. The *d* enantiomer of poly-lysine, poly-d-lysine (PDL) is often preferentially utilized over the *l* enantiomer, poly-l-lysine (PLL), due to its enhanced resistance to proteases such as trypsin (Li & Yeung, 2008; Stahmann et al., 1956), which improves the stability of the coating in long-term cultures. In contrast, neuronal cells derived from induced pluripotent stem cells (Takahashi & Yamanaka, 2006) are not sufficiently supported by a polycationic polymer coating alone. To adhere and survive these cells require an additional layer of Matrigel (Chambers et al., 2009) or a layer of laminin after coating over an initial layer of PLO (Perrier et al., 2004; Shi et al., 2012).

Here, our findings demonstrate that surfaces coated with dendritic polyglycerol amines (dPGAs) are significantly more effective and more stable than PLL or PDL. dPGAs are a family of polymers with a highly branched dendritic structure composed of glycerol monomers with a portion of the glycerol hydroxyl end groups substituted with amine functional groups (∼95 kDa with 50% amine functionalization) (Hellmund et al., 2015). dPGA presents a high density of positive charges at neutral pH, but unlike poly-lysine and poly-ornithine, does not contain peptide bonds and is therefore highly resistant to proteolysis (Frey and Haag, 2002). These polymers are relatively inexpensive to synthesize, easy to use and have a long shelf-life (Calderon et al., 2010). We report that dPGA functions as an enhanced long-term cell culture substrate coating that promotes the adhesion and differentiation of a variety of neural cells, including neurons derived from human iPSCs.

## Materials and Methods

### Coating Cell Culture Substrates

In brief, ∼95 kDa dPGA with 50% amine functionalization, was synthesized using an anionic polymerization of glycidol in dioxane to obtain dPG, followed by mesylation of 50% of the hydroxyl groups, azidation with sodium azide in DMF and Staudinger reduction using triphenyl phosphine and water. The final compound was purified using dialysis with cellulose membranes as described (Hellmund et al., 2015). For experiments using dPGA-coated substrates, round 12 mm diameter Deutsche Spiegelglas borosilicate glass #1 coverslips (Carolina Biological Supply, Burlington, NC, USA) were sterilized and cleaned using a plasma cleaner (Plasma Harricks) for 1 min with atmospheric gas mixture and then incubated overnight in the wells of a 24-well polystyrene cell culture plate (Corning) in 500 µl of a sterile solution of 1, 10, or 100 µg/ml of 150–300 kDa PDL (Sigma Aldrich, catalog number P1149) or dPGA in PBS in a cell culture incubator at 37° C with 5% CO_2_. Coated coverslips were then washed 3 ×  with PBS before neurons were seeded the same day. When a coating of laminin-1 was added to the cationic polymer coating, coverslips were incubated with an additional solution of 1 μg/ml of laminin-1 (α1β1β1 subunits) isolated from Engelbreth-Holm-Swarm murine sarcoma basement membrane (Sigma) for 1–2 h at 37° C and then washed 3 times with sterile PBS.

### Embryonic rat Cortical Neuron Culture

All animals were group-housed and provided *ad libitum* access to food and water. Cell cultures were prepared from cerebral cortices of embryonic day 18 (E18) Sprague Dawley rats as described (Banker & Goslin, 1998). Briefly, cortices from multiple embryos were pooled and dissociated cells plated at a density of ∼25,000 cells/cm^2^ in Dulbecco's Modified Eagle Medium with 10% fetal bovine serum and 1% Pen-Strep for 2 h to allow for cell adhesion before switching to Neurobasal medium containing 1% B27, 2 mM glutamax and 0.5% N2. All cultures were maintained for 7–90 days *in vitro* (DIV) at 37°C in a humidified 5% CO_2_ incubator. 50% of the media was changed every 7 days (all reagents for which a source is not listed were obtained from Thermo Fisher Scientific, CA).

### Immunocytochemistry

For immunolabeling, cells were fixed in 4% PFA for 12 min at room temperature, with the exception of labeling for synaptic markers, in which case cells were fixed in 100% MeOH for 8–10 min at −20° C. Cells were then blocked using 10% horse serum (HS), 3% bovine serum albumin (BSA) in PBS with 0.3% Triton X-100 (Sigma-Aldrich) for 90 min at rt. Cells were incubated overnight with primary antibodies in blocking solution (10% HS, 3% BSA and 0.3% Triton X-100 in PBS). The following antibodies and dilutions were used: rabbit anti-NFM 1:1000 (Millipore), mouse anti-S-100β 1:500 (Sigma Aldrich), rabbit anti-tyrosine-hydroxylase 1:1000 (Chemicon), mouse anti-tubulin β3 1:500 (BioLegend), mouse anti-NeuN 1:500 (Chemicon), guinea-pig anti-Synaptophysin-1 1:1000 (Synaptic System), mouse anti-PSD-95 1:500 (Thermo Fisher). After washing 3 × 15 min in PBS, cells were incubated with appropriate secondary antibodies: donkey anti-rabbit IgG Alexa 488 (Invitrogen), goat anti-guinea-pig IgG Alexa 555 (Invitrogen), donkey anti-mouse IgG Alexa 674 (Invitrogen) at a 1:500 dilution. Nuclei were labeled with the dye Hoechst 33342. Following labeling, cells were washed 3 × 15 min in PBS and then mounted using ProLong Gold.

Cells were imaged with a Leica SP8 confocal microscope using a 0.75 NA 20X objective (Leica Microsystems). Images were analyzed via custom scripts in imageJ (NIH). Briefly, Hoechst stained nuclei were segmented automatically and a mask was generated to count the number of labeled nuclei surrounded by a cytoplasm positive for a cell-type-specific marker. Statistical tests were performed using custom Python scripts.

### Electrophysiological Analysis

Whole cell patch-clamp recordings were made from embryonic rat cortical neurons (21 DIV) prepared as described previously (Glasgow et al., 2018). Neurons were plated on polymer-coated glass coverslips at a density of ∼50,000 cells/cm^2^ and incubated in BrainPhys medium (StemCell Technologies) supplemented with B27 (Bardy et al., 2015). Individual coverslips were transferred to an upright SliceScope 2000 microscope (Scientifica), and perfused with artificial cerebrospinal fluid (ACSF) containing (in mM): 135 NaCl, 3.5 KCl, 2 MgCl_2_, 2 CaCl_2_, 10 HEPES, and 20 D-Glucose (pH: 7.4, 290–300 mOsm). Single neurons with pyramidal-like morphology were targeted using differential interference contrast optics (40 ×  objective, 0.8 NA). Whole-cell recordings were made using borosilicate glass pipettes pulled with resistances of 4–9 MΩ and filled with (in mM): 120 K-gluconate, 20 KCl, 10 HEPES, 7 phosphocreatine di-Tris, 2 MgCl_2_, 0.2 ethylene-glycol-bis(b-aminoethyl ether)-N,N,N’,N’-tetraacetic acid (EGTA), 4 Na-ATP, 0.3 Na-GTP (pH: 7.2–7.26, 275–290 mOsm). Recordings were accepted if series resistance was <30 MΩ, which was monitored throughout the duration of the recording. Signals were amplified using a Multiclamp 700B amplifier and digitized using a Digidata 1550A (Molecular Devices). Current-clamp recordings were sampled at 20 kHz and filtered at 10 kHz, whereas voltage-clamp recordings were sampled at 10 kHz and filtered at 2 kHz using pClamp (v10.4, Molecular Devices).

Electrophysiological characteristics of hippocampal neurons were analyzed using Clampfit 10 software package (Molecular Devices). Spike properties were derived from the first action potential evoked in response to a 500-ms cathodal current injection. Spike amplitude was calculated based on resting membrane potential, whereas afterhyperpolarization and width were determined relative to spike threshold (>100 mV/ms). Input resistance was determined by the peak voltage response to a 500 ms, −100 pA anodal current injection from a holding voltage of −60 mV.

Spontaneous excitatory postsynaptic currents (sEPSC) were recorded in voltage-clamp mode using a holding potential of −70 mV, whereas spontaneous inhibitory postsynaptic currents (sIPSC) were recorded at a holding potential of 0 mV. sEPSC and sIPSC recordings were analyzed using MiniAnalysis (Synaptosoft) and individual synaptic events were detected using a threshold of 7 pA and 10 pA, respectively (>3 pA root mean square (RMS) of baseline current noise). Group averages were calculated based on all detected events. Cumulative distribution plots were generated using 100 randomly selected events per cell, which were subsequently rank-ordered.

Statistical analyses on parametric data were assessed using independent samples t-test, and normality, homoscedasticity, and outlier tests were performed on all datasets. For sEPSC and sIPSC cumulative distribution data, comparisons were performed using the Kolmogorov-Smirnov test. All electrophysiological data are presented as mean ± SEM, and data were analyzed and/or plotted using pClamp 10 suite (v10.4, Molecular Devices), MATLAB (Mathworks), Minianalysis (Synaptosoft), Prism 8 (GraphPad), Adobe Illustrator 6 (Adobe), and Sigmaplot 11 (Systat).

### Human iPSCs

#### Generation and maintenance of hiPSC

iPSCs were derived from human fibroblasts using a Cyto-Tune Sendai reprograming kit (Invitrogen) according to the manufacturer's instructions. iPSC colonies were cultured on Matrigel-coated dishes (BD Biosciences) using mTeSR1 medium (StemCell Technologies) changed daily.

#### Differentiation of cortical and dopaminergic neurons from iPSCs

Cortical and dopaminergic neurons were differentiated from hiPSCs following a modified version of previously described protocols (Chambers et al., 2009. The mTeSR media was substituted with DMEM/F12 supplemented with 1 ×  Glutamax, non-essentials amino acids, 15% KOSR, 1 × N2 and 1 × B27 supplements (Life Technologies), 100 ng/ml FGF8 (StemCell Technologies), 1 μM dorsomorphin (Tocris) and 1 μM SB431542 (Stemgent) and cultured for 1 week. hiPSC colonies were lifted off, cultured in suspension for 5 days to form embryoid bodies (EBs). EBs then were dissociated with accutase (Sigma), plated on a matrigel-coated dish and fed with expansion media composed of DMEM/F12 supplemented with 1X N2 supplement, 1X B27 supplement (Life Technologies), 1% penicillin/streptomycin, and 20 nM FGF2 (Prepotech) and 10 nM EGF (Prepotech). Emerging rosettes were dissociated completely using accutase and plated on poly-ornithine (50 ug/ml)/laminin (1 ug/ml) or dPGA (50 ug/ml)/laminin (1 ug/ml)-coated dishes (Sigma). NPCs were expanded and fed every 2 days.

To generate cortical neurons, NPCs were differentiated using Neurobasal media containing, 1X Glutamax, 1X B27, 1X N2, 20 ng/ml BDNF (Prepotech), 20 ng/ml GDNF (Prepotech), 1 ng/ml Compound E (Calbiochem) and 1 ng/ml VPA (Sigma). To generate midbrain dopaminergic neurons, NPCs were differentiated using Neurobasal media containing, 1X B27, 1X N2, 20 ng/ml BDNF (PrepoTech), 20 ng/ml GDNF (PrepoTech), 1 ng/ml compoundE (Calbiochem), 0.2 mM ascorbic acide (Sigma), 0.5 mM dbcAMP (Carbosynth), 1 ng/ml TGF3β (Prepotech).

#### Differentiation of hippocampal neurons from iPSCs

All iPSCs were characterized as previously described (Brennand et al., 2011). EBs were formed by mechanical dissociation of iPSC colonies using collagenase and plating onto low-adherence dishes. For EB differentiation, floating EBs were treated in STEMdiff™ Neural Induction Medium + SMADi (StemCell Technologies) for 20 days. To obtain neural progenitor cells, EBs were then plated onto polyornithine/laminin (Sigma)-coated dishes in DMEM/F12 plus 1% N2 and 1% B27. Rosettes were manually collected and dissociated with accutase (Chemicon) after 1 week and plated onto PLO (50 µg/ml) and laminin-coated dishes in neural progenitor cell media (DMEM/F12, 1% N2, 1% B27 (Invitrogen), and 20 ng/ml  EGF, and 20 ng/ml  FGF2 (Invitrogen)). To obtain hippocampal mature neurons, neural progenitor cells were plated onto dPGA or PLO (50 µg/ml) and laminin (1 µg/ml) coated-dishes and differentiated in BrainPhys neuronal medium (StemCell Technologies) supplemented with 1 × N2, 1 × B27, 20 ng/ml BDNF (Peprotech), 1 mM dibutyrl-cyclicAMP (Sigma), 200 nM ascorbic acid (Sigma), 1 μg/ml laminin and 620 ng/ml Wnt3a (R&D) for 2 weeks. After 2 weeks post differentiation, media was replaced by STEMdiff™ Forebrain Neuron Maturation Kit (StemCell Technologies) in BrainPhys media until use. All cells used in the present study were verified as free from mycoplasma contamination.

#### Atomic force microscopy

All AFM images were acquired in liquid (DI water) with tapping mode on an Asylum MFP-3D-Bio AFM (Oxford Instruments-Asylum Research, Santa Barbara, USA). Tapping mode cantilevers with a resonant frequency of 70 kHz were used (240AC-PP manufactured by NANOANDMORE USA). Images were acquired by oscillating the cantilevers at their 1^st^ resonant frequencies and parameters were optimized so that very minimal force was applied to avoid tip-induced sample modifications. The resulting images were then processed and the RMS roughness value extracted in Gwyddion software (post-2.52 development version) (Nečas, 2012).

#### Statistical analysis

Statistical analysis was performed using the SciPy python library (version 1.6.3) via custom scripts. The Shapiro–Wilk test was used to test the normality of the raw data. Comparison of the means between cultures grown on dPGA versus PDL/PLO were performed within experiments by Student's t-test for appropriate pairs.

## Results

### dPGA-Substrate Coating Supports Primary Mammalian Neuronal Cells in Culture

**Figure 1.**
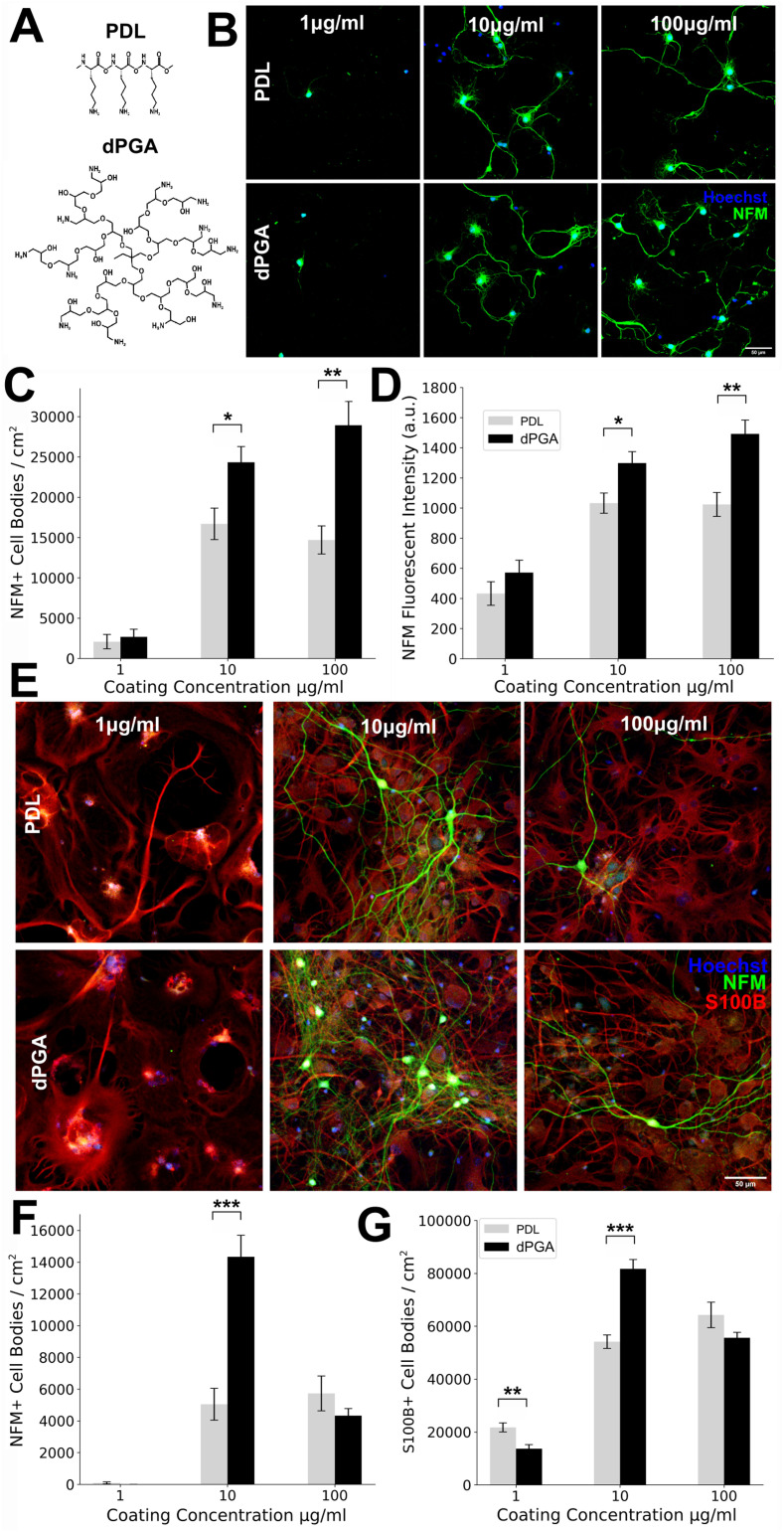
Enhanced survival of neocortial neurons grown on dPGA-coated surfaces compared to a standard PDL coating. (A) Schematic illustrations of PDL and dPGA polymers. (B) E18 rat neocortical neurons at 7 DIV grown on increasing concentrations of either PDL or dPGA coated glass coverslips immunolabeled for neurofilament M (NFM) and stained with Hoechst dye 33342 to label nuclei (scale bar is 30 µm). (C) Quantification of NFM positive cell density. (D) NFM fluorescent intensity in primary cultures of E18 rat neocortical neurons at 7 DIV maintained on glass coverslips treated with increasing concentrations of PDL or dPGA (*p < .05, ** *p* < .01, paired two-tailed Student's *t*-test n = 4). (E) E18 rat neocortical cultures following 90 DIV immunolabeled for neuronal marker NFM, glial marker S100β, and nuclear dye Hoechst 33342 (scale bar 50 µm). (E-G) Quantification of NFM positive cell density (E) and S100β positive cell density (G) detected following 90 DIV on glass coverslips treated with increasing concentration of either PDL or dPGA (*p < .05, **p < .01, ***p < .001, paired two-tailed Student's *t*-test n = 10).

Substrates coated with poly-cationic peptides are widely employed to support the adherence and survival of neural cells in culture. Poly-lysine is the most commonly used polymer for primary neuronal culture (Banker & Goslin, 1998), however peptide-based cell culture coatings are sensitive to proteolysis and are degraded by cells *in vitro*. We therefore set out to determine if a non-peptidergic poly-cationic polymer might provide enhanced support for long-term cell culture. Here we tested primary neurons seeded on a cell culture surface coated with ∼95 kDa dPGA with ∼50% of the terminal hydroxyl groups converted to amines, compared to a standard poly-lysine coating (Figure 1A). Our comparison focused on PDL, the D enantiomer of poly-lysine, due to its relative resistance to trypsin-like proteases (Li & Yeung, 2008) compared to L polymers such as PLL or poly-L-ornithine (PLO) coatings.

A cell culture substrate, such as a clean glass coverslip or TC-treated polystyrene petri dish, is typically coated by adsorption from a PDL solution in the range of 10–100 µg/ml. Here, we compared plasma-cleaned glass coverslips coated with concentrations of 1, 10 and 100 µg/ml of PDL or dPGA in sterile PBS, testing the typical range of concentrations used as a coating solution. Although coating for 1 h is generally sufficient and previous studies have concluded that most polymer adsorption occurs within the first 5–10 min (Choi et al., 2015; Kosior et al., 2020), we incubated the culture surface with the coating solution overnight at 37° C to preclude any difference in coating efficiency due to possible discrepancies in the adsorption kinetics between the two polymers. Following adsorption, the substrates were washed with PBS.

To evaluate whether dPGA coated surfaces could support the adhesion and survival of neuronal cells *in vitro*, we seeded the coated coverslips with dispersed primary neocortical neurons derived from embryonic day 18 (E18) rat cortices. We found that glass coverslips coated with 0.5 ml solution of PDL or dPGA at 10 or 100 µg/ml were sufficient for primary cortical neurons to adhere to the culture surface within 1 h after seeding and that both of these surfaces similarly supported neuronal survival and neurite extension for several days afterward (Figure 1B–D).

Following 7 days *in vitro* (DIV), cultures were fixed and labeled with antibodies against the neuron-specific medium molecular weight neurofilament subunit (NFM), and the glial specific marker S100β. The number of cells positive for these markers was counted, providing an assessment of the overall health and composition of the cultures. Our findings revealed an ∼52% higher density of neurons grown on substrates coated with 10 µg/ml dPGA compared to 10 µg/ml PDL (16,691 ± 3,902 for PDL vs. 24,333 ± 3,892 cells/cm^2^ for dPGA, *p* < .05, Student's t-test, n = 8 independent cultures), and an ∼96% higher neuronal density on substrates coated with 100 µg/ml dPGA compared to 100 µg/ml PDL (14,689 ± 3,482 for PDL vs. 28,932 ± 5,908 cells/cm^2^ for dPGA, *p* < .01, Student's t-test, n = 8 independent cultures) (Figure 1C). In accordance with the increased cell density, we measured a significant increase in the overall NFM fluorescence intensity in cultures grown on a dPGA coating versus a PDL coating (∼25% and ∼46% increase of dPGA vs. PDL, *p* < .05 and *p* < .01 at a concentration of 10 and 100 µg/ml respectively, Student's t-test, n = 8 independent cultures) (Figure 1D).

Unlike the substrates coated with 10 or 100 µg/ml PDL or dPGA, coatings using 1 µg/ml of either polymer resulted in few surviving neurons, and the limited number of cells detected exhibited a stunted morphology with minimal neurite extension and extensive surface blebbing. These findings indicate that coating a glass substrate with a solution of 1 µg/ml of either PDL or dPGA, using the methodology detailed here, is insufficient to maintain healthy adherent cortical neurons in culture. Examination of the culture via phase-contrast imaging before fixation showed a similar sparse distribution of surviving neurons, indicating that the few cells present after immunolabeling are not due to cellular detachment during the fixation, washing and labeling steps, but rather reflect the inability of cells to adhere and survive on a minimally coated glass surface. Certain polycationic surface coatings may impact the proliferation or differentiation of either neurons or glia (Ji et al., 2019). However, few S100β positive glial cell bodies were present in these 7 DIV cultures, and the few cells detected did not appear to be significantly affected by the type of substrate used (data not shown).

### dPGA Supports Primary Cortical Neurons in Vitro for at Least 3 Months

PDL is resistant to proteolysis by trypsin but is likely susceptible to degradation by other proteases (Stahmann et al., 1956). In contrast, the polyglycerol core of dPGA entirely lacks peptide bonds and is highly resistant to degradation by cellular proteases (Calderon et al., 2010). In view of the increased neuronal density observed at 7 DIV on dPGA coated substrates and its insensitivity to proteolysis, we investigated whether a substrate coated with dPGA might support more stable longer-term cell cultures. To test this, we maintained cultures of E18 rat primary cortical neurons grown on either a dPGA or PDL coated substrate for 90 DIV (3 months). In all conditions, fewer neurons were detected compared to 7 DIV, consistent with neuronal density typically decreasing over the first 2 months of culture (Harrill et al., 2015; Lesuisse & Martin, 2002). The neurons remaining appeared healthy with large cell bodies, extensive arborisations (Figure 1E) and synaptic connections (Supplementary Figure 1). Quantification revealed that the number of neurons supported by the dPGA coated substrate was ∼3-fold more compared to the PDL substrate (5,045 ± 3,162 for PDL vs. 14,333 ± 4,307 cells/cm^2^ in dPGA vs. PDL, *p* < .001, Student's t-test, n = 10 independent cultures) (Figure 1F). We detected a substantial increase in the density of glial cells at 3 months *in vitro* compared to 7 DIV. S100β positive cells with astrocyte-like morphology formed a largely confluent layer. Slightly more S100β positive cells were detected on the 10 µg/ml dPGA-coated substrate compared to 10 µg/ml PDL-coated substrate, but no significant difference in glial cell density was found when the substrates were coated with 100 µg/ml (Figure 1G). Notably, the enhanced capacity of the dPGA substrate to support neurons was apparent only at a coating concentration of 10 µg/ml. Fewer neurons were detected in cultures coated with 100 µg/ml of dPGA compared to the 10 µg/ml dPGA coating, with neuronal and glial cell densities that were not significantly different from the cultures coated with 100 µg/ml of PDL (5,727 ± 3,469 for PDL vs. 4,332 ± 1,407 cells/cm^2^ for dPGA, *p* > .05, Student's t-test, n = 10 independent cultures).

### Primary Neocortical Neurons Differentiate, Polarize, and Elaborate Synaptic Connections on a dPGA Coated Substrate

**Figure 2.**
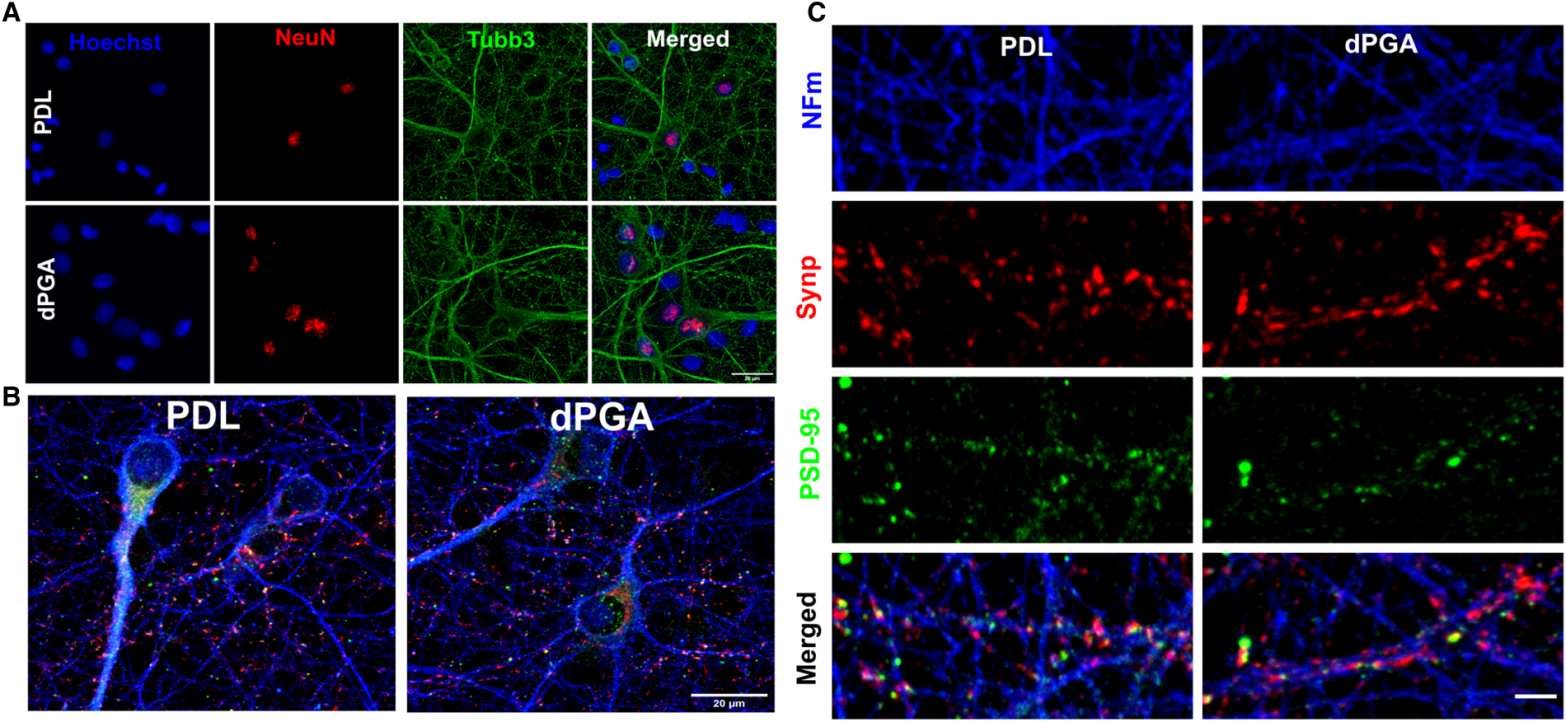
Differentiation of primary neocortical neurons cultured on dPGA. (A) E18 rat neocortical neurons at 12 DIV on coverslips coated with 100 µg/ml of PDL or dPGA and immunolabled for neuronal markers NeuN and β3-Tubulin (tubb3, scale bar 20um). (B) E18 rat neocortical cultures at 12 DIV on coverslips coated with 100 µg/ml of either PDL or dPGA and stained with neuronal specific NFM, and synaptic markers Synaptophysin-1 (Synp) and PSD-95 (scale bar 20um). (C) E18 rat neocortical neurons at 12 DIV on coverslips coated with 100 µg/ml of PDL or dPGA and immunolabeled for NFM, presynaptic Synaptophysin-1 and postsynaptic PSD-95 (scale bar 2 µm).

The findings described above provide evidence that a dPGA coated substrate improves neuronal survival in culture, however it was not known how this novel material may impact neuronal differentiation and function *in vitro*. We therefore tested for possible differences between neurons grown on PDL or dPGA. Cells were immunocytochemically labeled using the neuron-specific markers beta III Tubulin (tubb3), NFM, and the neuronal nuclear marker NeuN. Examining cells grown on dPGA or PDL coated substrates at 12 DIV we found similar neuronal polarization and process elaboration (Figure 2A).

A hallmark of neuronal differentiation is the capacity to form synapses and propagate electrophysiological signals. Examining cultures grown on PDL or dPGA substrates following 12 DIV, we immunolabeled for the presynaptic marker synaptophysin-1 and glutamatergic postsynaptic marker PSD-95 and evaluated the distribution of these proteins along neurites. Embryonic rat cortical neurons grown on substrates coated with 10 µg/ml of either dPGA or PDL exhibited similar immunoreactivity for synaptophysin-1 and PSD95 (Figure 2B), with punctate enrichments distributed along neurites. Partially overlapping puncta enriched for each of the two proteins were readily detected along adjacent neurites, consistent with pre- and post-synaptic specializations (Figure 2C). Although a dPGA coated substrate increases neuronal survival in long-term culture, as described above, no differences were readily apparent in the intensity or distribution of synaptic markers along neurites between cultures grown on substrates coated with PDL compared to dPGA (data not shown).

### Electrophysiological Properties of Neocortical Neurons Grown on dPGA Coated Substrate do not Differ from the Properties of Neurons Grown on PDL

**Figure 3.**
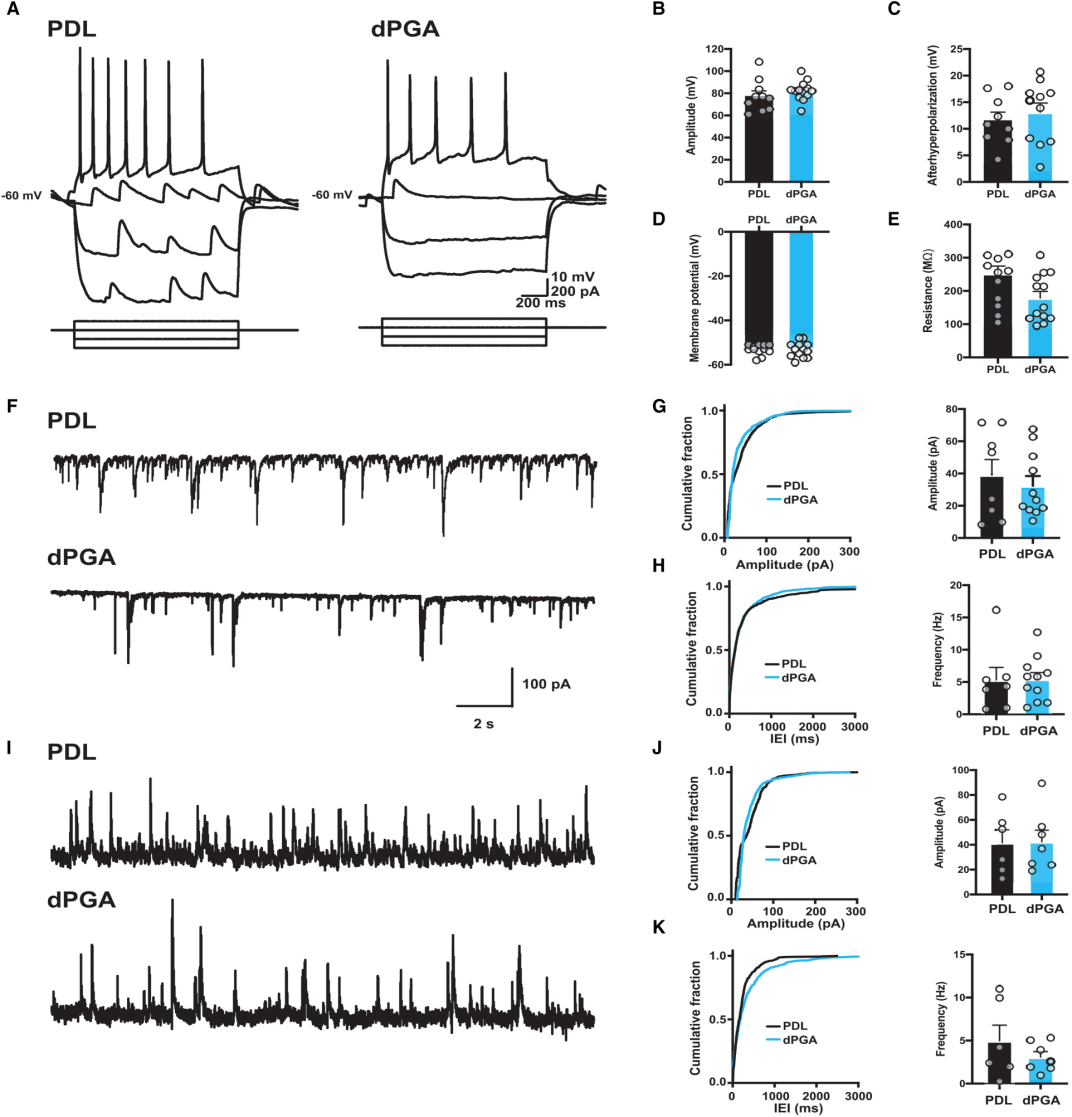
Similar electrophysiological properties of primary cortical neurons grown on dPGA-coated and PDL-coated surfaces. (A–E) Intrinsic excitability of primary rat neocortical neurons was not altered when grown on a dPGA versus a standard PDL-coated substrate. (A) Membrane voltage responses to a series of hyperpolarizing and depolarizing intracellular current pulses in neurons grown on PDL-coated (left) and dPGA-coated (right) substrates. Group data show no significant differences between neurons grown on PDL-coated (black) and dPGA-coated (blue) substrates in (B) action potential amplitude, (C) afterhyperpolarization amplitude, (D) resting membrane potential, or € input resistance. (F–K) Synaptic transmission is maintained in neurons grown on PDL-coated and dPGA-coated substrates. (F) Representative current traces of sEPSCs from neurons grown on a PDL-coated (top) and dPGA-coated (bottom) substrate with a holding potential of −70 mV. Cumulative distribution plots (left) and group data (right) show no significant differences in sEPSC amplitude (G; PDL: 39.1 ± 9.6 pA vs. dPGA: 32.4 ± 6.0 pA, t17 = 0.62, *p* = .54 [unpaired t-test]) and frequency (H; PDL: 5.3 ± 1.9 Hz vs. dPGA: 5.4 ± 1.1 Hz, t17 = 0.05, *p* = .95 [unpaired t-test]) recorded in neurons grown on PDL-coated (black) and dPGA-coated substrates (blue). n = 8 cells in PDL, 11 cells in dPGA. (I) Representative current traces of sIPSCs from neurons grown on a PDL-coated (top) and dPGA-coated (bottom) substrate with a holding potential of 0 mV. Cumulative distribution plots (left) and group data (right) show no significant differences in sIPSC amplitude (J; PDL: 41.6 ± 10.4 pA vs. dPGA: 42.4 ± 9.3 pA, t11 = 0.06, *p* = .95 [unpaired t-test]) and frequency (K; PDL: 4.9 ± 1.8 Hz vs. dPGA: 3.1 ± 0.6 Hz, t11 = 1.04, *p* = .32 [unpaired t-test]) recorded in neurons grown on PDL-coated (black) and dPGA-coated substrates (blue). n = 6 cells in PDL, 7 cells on dPGA.

We then compared the electrical properties of embryonic rat primary neocortical neurons maintained on dPGA or PDL coated substrates. Whole cell patch-clamp recordings revealed that primary neocortical neurons grown on glass coverslips coated with 10 µg/ml dPGA generated electrophysiological activity and synaptic events that were not distinguishable from cultures maintained in parallel on a standard PDL-coated surface (Figure 3). Neocortical neurons grown on dPGA coated or PDL-coated glass showed no significant difference in intrinsic cellular properties (Figure 3A), including action potential amplitude (Figure 3B) and afterhyperpolarization (Figure 3C). Moreover, we detected no significant differences in resting membrane potential (Figure 3D), or input resistance (Figure 3E) between neurons plated on dPGA- or PDL-coated glass.

Consistent with our immunocytochemical visualization of synaptic markers (Figure 2), voltage clamp recordings revealed spontaneous synaptic potentials indicating functional synaptic transmission (Figure 3F–K). We observed no significant differences in excitatory postsynaptic current (EPSCs) amplitude or frequency between the different substrates. Similarly, we observed no significant differences in inhibitory postsynaptic currents (IPSCs) between conditions. These findings support the conclusion that dPGA compared to PDL is a superior substrate to support and maintain short and long-term cultures of primary cortical neurons, without significantly altering neuronal physiology.

### dPGA Substrate Enhanced Cell Density due to Maturation and not Initial Adhesion

**Figure 4.**
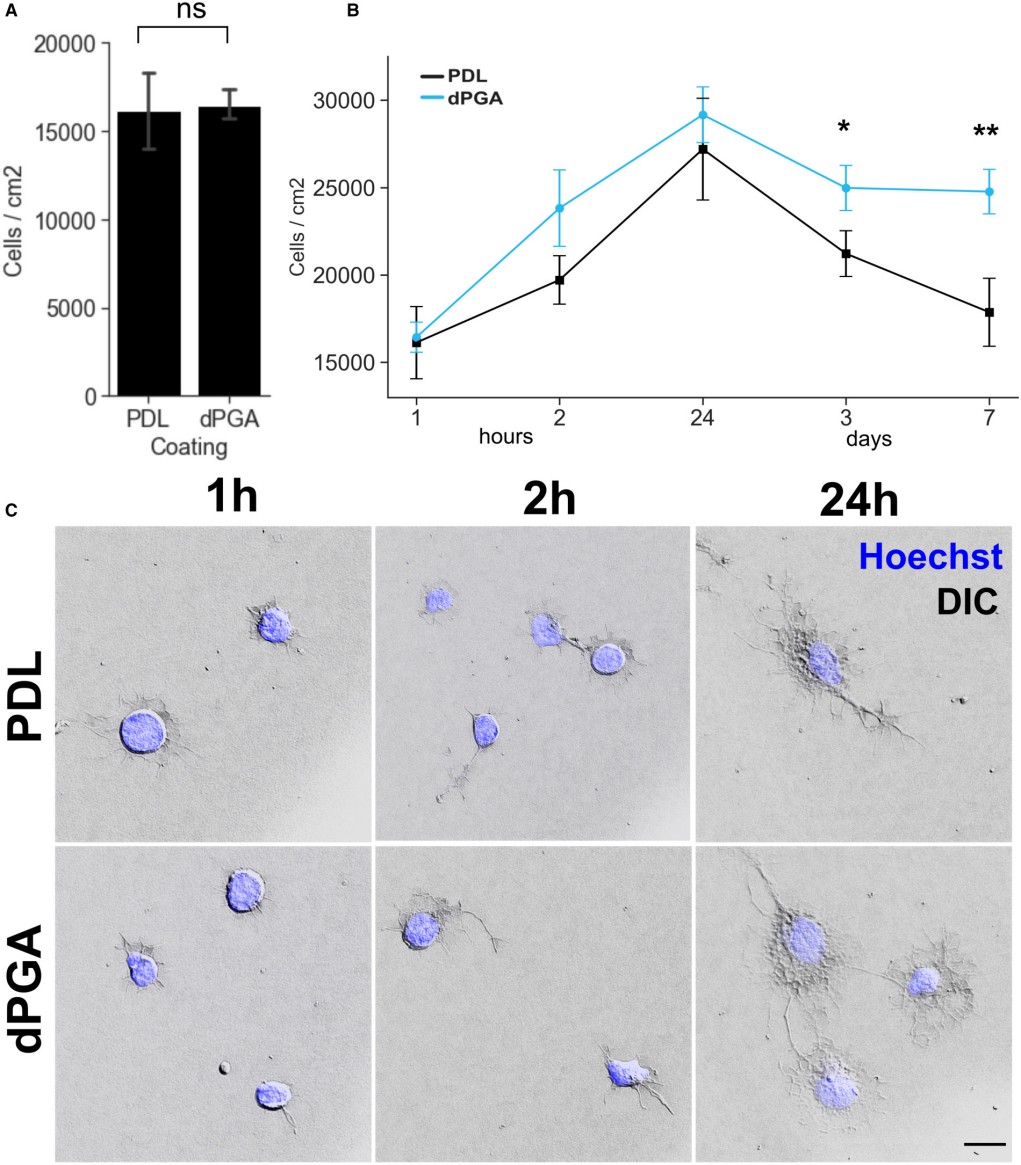
Increased density of primary cortical neurons on dPGA-coated substrate becomes apparent after 3 DIV. (A) No significant difference was detected in the number of cells adhering to PDL or dPGA-coated glass coverslips 1 h post-seeding E18 rat neocortical neurons (*p* = 0.89, paired two-tailed Student's *t*-test n = 12). (B) Measurement of cell density over a 7-day time course indicates that differences in the number of cells adhering to PDL vs dPGA coated subtrates becomes significant 3 days after plating (*p < .05, **p < 0.01, paired one-tailed Student's t-test n = 12-16). All values are normalized to the 1 h PDL value. (C) DIC images shows similar morphologies of primary neuronal cells within the first 1, 2, and 24 hs of seeding and adheering onto a PDL or dPGA-coated substrate (10 µg/ml solution). Imaged using DIC optics, 63 ×  objective, and overlayed with Hoechst 33342 fluoresence to visualize nuclei (scale bar 5 µm).

A straightforward explanation for the higher number of neurons that we detected on dPGA versus PDL coated substrates may be that more cells may initially adhere to the surface during the plating process before the initial media change 2 h after plating. To determine if this was the case we examined the composition of the cultures at 1 and 2 h post-plating, before the initial full media change. No significant difference was found between the number of cells adhered to either substrate (Figure 4A). These findings suggest that the difference detected is not due to more cells initially adhering to dPGA compared to PDL, but likely to later events in the culture.

In addition to adhesive mechanisms that mechanically tether the cells to the substrate, the substrate composition can also impact cellular proliferation, differentiation, growth and survival. To better understand the mechanism underlying the capacity of dPGA to support cells, we examined cell counts of sister cultures fixed after various time points following the initial cell plating to determine when cell densities initially diverge between dPGA and PDL (Figure 4B). For both substrates, we found that cell density increased between 1 h post-plating and 24 h, followed by a decline between 24 h and 3 days, however, the cell density in PDL-coated cultures continued to decline between 3 and 7 days while the number of cells supported by a dPGA substrate remained relatively stable. Although the PDL-substrate cultures had a slightly lower mean cell density at all timepoints measured, no statistically significant difference in cell density (Figure 4B) or morphology (Figure 4C) was measured between PDL and dPGA coated substrates at 1 h, 2 h and 24 h post-plating. The significant difference in cell density only emerged after 3 days in culture, and became more apparent at 7 DIV as the number of remaining cells in PDL cultures continued to decline. These findings provide evidence that the larger number of neural cells supported by dPGA is not the result of increased initial cell adhesion but rather to an enhanced capacity of the substrate to maintain surviving cells.

### dPGA Substrate Supports Long-Term Human iPSC-Derived Cortical Neuron Cultures

The poly-cationic peptide PLO is also routinely used as a substrate coating for cell culture and, in particular, is often applied to support the differentiation and maintenance of iPSC-derived neuronal cells in feeder-free conditions. A major application for human iPSC-derived neurons is to study age related neurodegenerative diseases. These cells in particular, would benefit from protocols that enhance the stability of long-term cultures. We therefore explored the possibility of replacing PLO with dPGA as a cell culture substrate to enhance the stability of long-term cultures of human iPSC-derived neurons. A typical feeder-free configuration to support iPSC-derived neurons is the application of a base layer of PLO followed by a layer of laminin (Perrier et al., 2004; Shi et al., 2012), or a layer of Matrigel without a PLO base layer (Chambers et al., 2009).

We first tested whether dPGA could provide an economical alternative to replace substrate coatings of laminin or Matrigel. Human neural progenitor cells plated during the final differentiation step for cortical neurons onto substrates coated overnight with 50 µg/ml of dPGA or 50 µg/ml of PLO alone did not survive (data not shown). Like PLO, dPGA presents a positively charged electrostatic surface, but does not contain the specific receptor binding sites present in a protein like laminin-1 (Edgar et al., 1984; Simon-Assmann et al., 2011). We then tested whether dPGA might replace or enhance the foundation layer of PLO used in conjunction with the laminin layer. Glass coverslips coated overnight with 50 µg/ml of dPGA or 50 µg/ml of PLO were rinsed and incubated for an additional 2 h with a solution of commercially available laminin-1 (1 µg/ml) before seeding them with hiPSC-derived neural progenitor cells.

**Figure 5.**
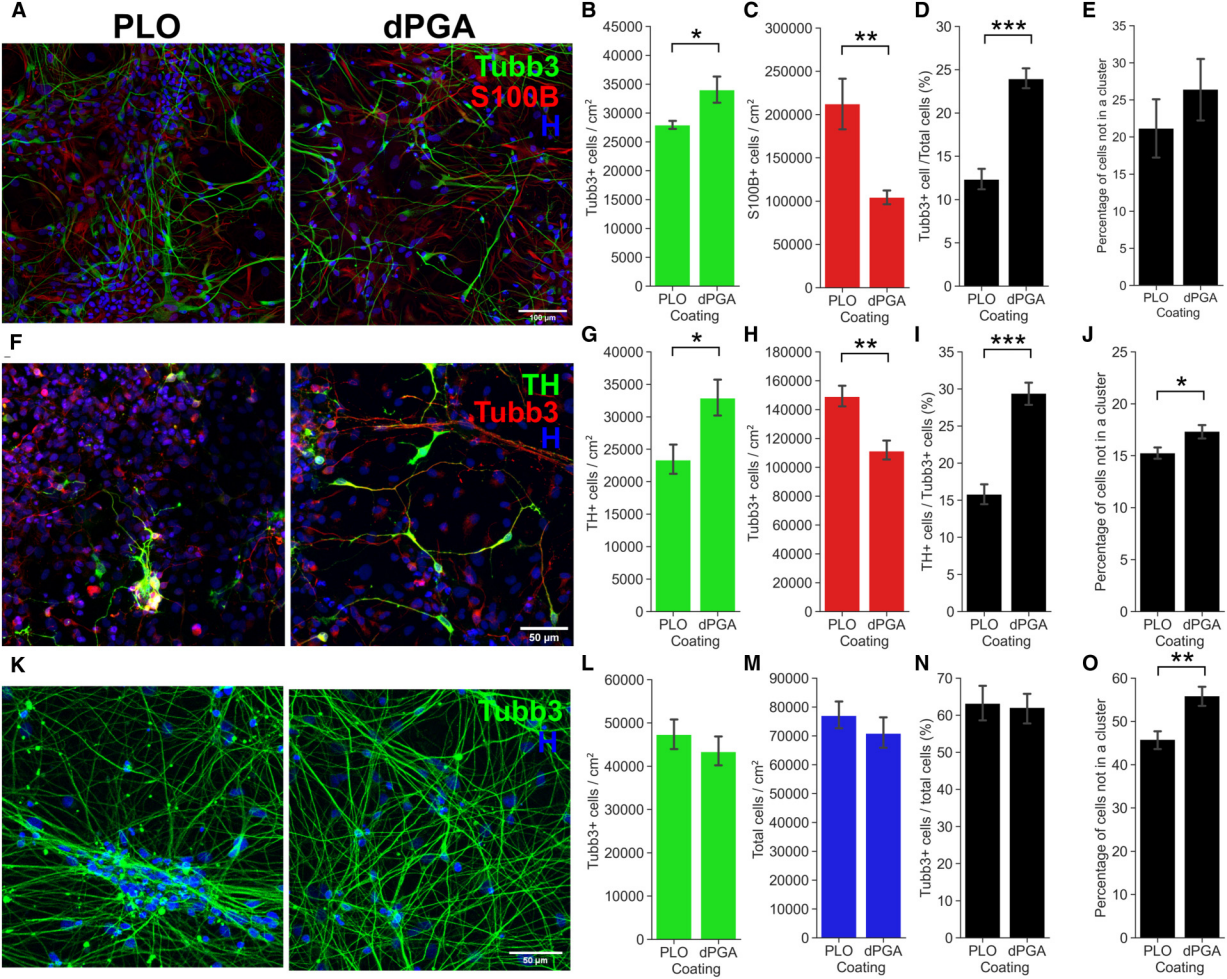
Enhanced survival and differentation of hiPSC-derived neuronal cultures grown on dPGA-laminin coated surfaces compared to standard PLO-laminin. (A-E) Comparison of the composition of hiPSC-derived cortical neuron cultures grown on dPGA-laminin versus PLO-laminin substrate coating. (A) 14 DIV hiPSC-derived cortical neuron cultures on PLO-laminin (left) and dPGA-laminin (right) coated surfaces, stained with Hoechst nuclear dye (blue) and immunolabled for neuron specific β3-Tubulin (Tubb3, green) and glial specific S100β (red) (scale bar 100 µm). (B-D) Quantification of the density of (B) β3-Tubulin positive cells and (C) S100β positive cells. (D) Percentage of β3-Tubulin positive neurons compared to the total number of cells. (E) Number of cells not clustered (defined as 2 cells or more less than 1 µm apart from each other) at 14 DIV in hiPSC-derived cortical neuron cultures on PLO-laminin or dPGA-laminin (* *p* < .05, **p < .01, ***p < .001, paired two-tailed Student's *t*-test n = 6). (F-J) Comparison of hiPSC-derived midbrain dopaminergic neuronal culture composition when grown on dPGA-laminin versus PLO-laminin. (F) 14 DIV hiPSC-derived dopaminergic neuron cultures on PLO-laminin (left) and dPGA-laminin (right) coated surfaces, stained with Hoechst dye (blue) and immunolabeled for tyrosine hydroxylase (TH, green) and β3-Tubulin (red, scale bar 50 µm). (G-I) Quantification of the density of (G) TH positive cells and (H) Tubb3 positive cells. (I) Percentage of TH positive neurons compared to the total number of Tubb3 positive neurons. (J) The number of cells not in a cluster in hiPSC-derived dopaminergic cultures at 14 DIV on PLO-laminin or dPGA-laminin coated substrates (*p < .05, **p < .01, ***p < .001, paired two-tailed Student's *t*-test n = 6). (K-O) Comparison of hiPSC-derived hippocampal neuron cultures on a dPGA-laminin versus PLO-laminin coated substrates. (K) Representative photomicrographs of 14 DIV hiPSC-derived hippocampal neuron cultures on PLO-laminin (left) and dPGA-laminin (right) coated surfaces, stained with Hoechst dye (blue) and immunolabled for β3-Tubulin (green) (scale bar 50 µm). (L) Quantification of the density of Tubb3 positive cells. (M) Density of all cells in culture. (N) Percentage of Tubb3 positive neurons compared to all cells. (O) Number of cells not in a cluster in 14 DIV hiPSC-derived dopaminergic neuron cultures on PLO-laminin or dPGA-laminin coated substrates (*p < .05, **p < .01, ***p < .001, paired two-tailed Student's t-test n = 16 and 20 respectively).

Following 1 week in culture, inspection with phase-contrast microscopy revealed that the cultures maintained on substrates of dPGA-laminin were healthier than cultures grown on PLO-laminin. Following 2 weeks, the hiPSC-derived cultures were immunolabeled for NFM and S100β, and nuclei stained with Hoechst dye. Quantification detected ∼21% more iPSC-derived cortical neurons on the dPGA-laminin coated substrate compared to cultures grown on the standard PLO-laminin substrate (Figure 5A) (27,899 ± 1,871 for PLO vs. 34,011 ± 6,415 cells/cm^2^ for dPGA, *p* < .05, Student's *t*-test, n = 6 independent cultures). Intriguingly, the total cell density, determined by counting Hoechst labeled nuclei, was lower in dPGA-laminin compared to PLO-laminin cultures. Immunostaining and quantification revealed that PLO-laminin cultures contained approximately twice as many S100β positive cells as the dPGA-laminin cultures (Figure 5B) (212,340 ± 77,773 for PLO vs. 104,448 ± 19,569 cells/cm^2^ for dPGA, *p* < .01, Student's t-test, n = 6 independent cultures). The higher neuron to glia ratio suggests a bias toward neuronal differentiation in hiPSC-derived cortical cultures grown on a dPGA-laminin substrate (Figure 5C–E) (12.3% for PLO vs. 23.9% of Tubb3 positive cells for dPGA, *p* < .001, Student's t-test, n = 6 independent cultures).

### dPGA Supports Other Types of Human iPSC-Derived Neurons

The results described above provide evidence that a layer of dPGA is a superior cell culture substrate compared to standard coatings for primary or iPSC-derived cortical neurons. To test the generalizability of dPGA and potentially extend its utility as a cell culture substrate for other neuronal cell types, we then tested its capacity to support human iPSC-derived dopaminergic neurons and hippocampal neurons. These neurons are of notable interest because of their potential to model human age-related neurodegenerative diseases, for example, when derived from Parkinson's disease and Alzheimer's disease patients (Israel et al., 2012; Soldner et al., 2009).

### dPGA Supports Human iPSC-Derived Dopaminergic Neurons

Following the final differentiation step toward midbrain dopaminergic neurons, neural progenitor cells were seeded using the same conditions as described above for iPSC-derived cortical neurons. After 14 DIV the cultures were immunolabeled for the dopaminergic neuronal marker tyrosine hydroxylase (TH), the general neuronal marker beta III Tubulin, and with Hoechst dye to label nuclei (Figure 5F). In these cultures, it is important to identify the dopaminergic neurons as current protocols for the generation of midbrain dopaminergic neurons from hiPSCs yield a mixture of dopaminergic and non-dopaminergic neurons (Soldner et al., 2009). Quantification of the different cell types indicated that the dPGA-laminin substrate resulted in significantly more TH-positive neurons compared to cultures grown on the PLO-laminin substrate (23,340 ± 9,068 for PLO vs. 32,881 ± 10,997 TH + cells/cm^2^ for dPGA, *p* < .05, Student's t-test, n = 15 independent cultures) (Figure 5G). Surprisingly, although more TH-positive neurons were detected, the dPGA-laminin condition also resulted in fewer total beta III Tubulin-positive neurons (148,935 ± 30,980 for PLO vs. 111,182 ± 26,711 TH + cells/cm2 for dPGA, *p* < .01, Student's t-test, n = 15 independent cultures) (Figure 5H) and fewer total cells overall (Hoechst positive nuclei) compared to the PLO-laminin substrate (data not shown). These findings support the conclusion that the dPGA-laminin substrate better supports differentiation toward the targeted dopaminergic neuronal cell type (Figure 5I).

### dPGA Supports Human iPSC-Derived Hippocampal Neurons

Examining hiPSC-derived hippocampal neurons, following the final differentiation step, the neural progenitor cells were similarly plated on glass coverslips coated with a combination of either dPGA-laminin or PLO-laminin as described above. After 28 days in culture, the cells were immunolabeled for the neuronal marker beta III Tubulin and nuclei stained with Hoechst dye (Figure 5K). We identified no significant differences in total number of cells, total number of beta III Tubulin positive neurons, or the percentage of cells that differentiated into hippocampal neurons (Figure 5L–N) between hiPSC-derived hippocampal neurons grown on a dPGA-coated surface compared to neurons plated on a standard PLO-coated surface (74,789 vs. 69,850 cells/cm^2^, 47,265 vs. 43,336 neurons/cm^2^, 63% vs. 62% percent of neurons to total cells for PLO-laminin vs. dPGA-laminin, *p* = .40, *p* = .24, *p* = .58 respectively, Student's *t*-test, n = 16 independent cultures). However, we did observe that hiPSC-derived hippocampal neurons tended to aggregate into large clusters over time in cultures grown on PLO-coated glass compared to cultures grown on dPGA-coated glass. Quantification of the number of beta III Tubulin positive cells found within a cluster (defined as 2 or more beta III Tubulin positive neurons located less than 1 µm apart) revealed a significant increase in hippocampal neurons clustering in cultures that were seeded on PLO-coated surfaces compared to dPGA-coated glass after 28 DIV (Figure 5O) (56% for PLO-laminin vs. 46% for dPGA-laminin, *p* < .01, Student's *t*-test, n = 16 independent cultures). A similar, albeit modest, tendency for reduced cell aggregation was detected in hiPSC-derived midbrain dopaminergic neuronal cultures (Figure 5J). These findings support the conclusion that dPGA-laminin is a more stable substrate for the long-term maintenance of cultured hiPSC-derived neurons.

### Increased Nano-Scale Roughness of dPGA Coated Substrate Compared to Poly-Lysine

**Figure 6.**
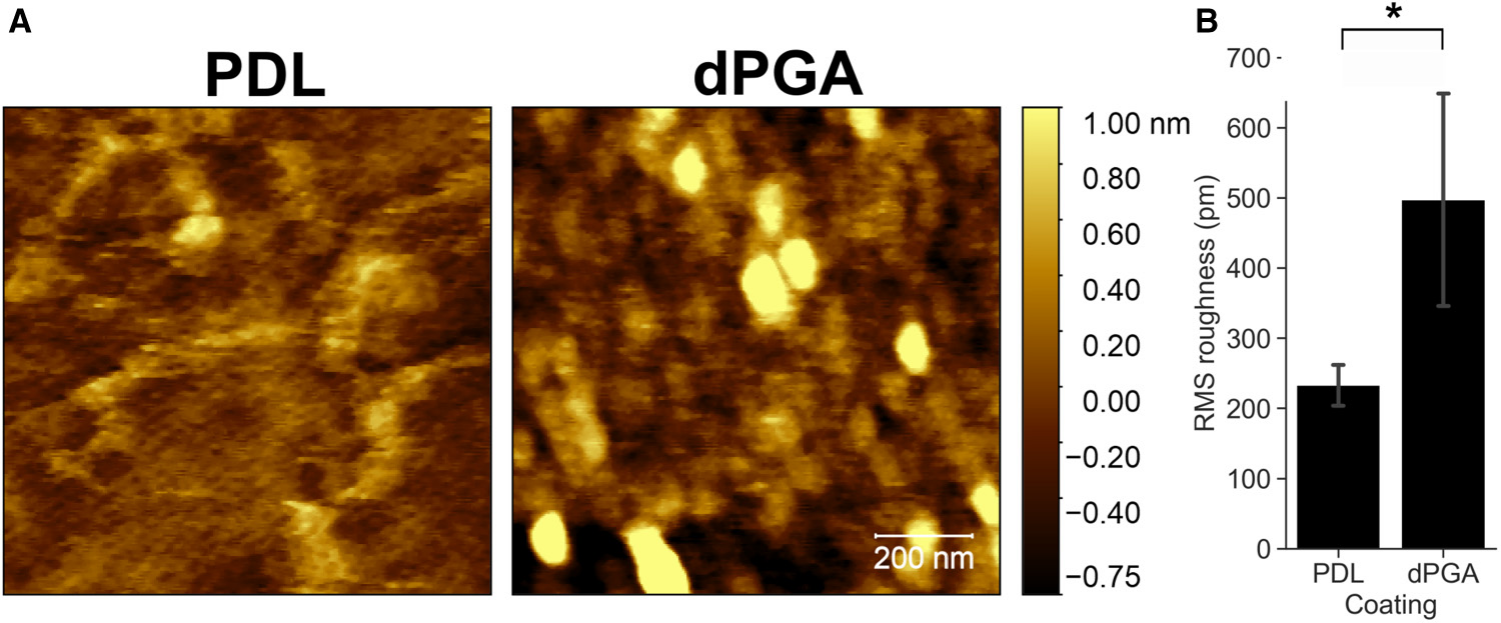
Nanotopological characteristics of dPGA and PDL coated substrates. Borosilicate glass coverslips were coated with 10 µg/ml solutions of either PDL or dPGA. (A) Surface topography imaged with AFM (scale bar 200 nm). (B) RMS roughness determined by AFM (*p < .05, paired one-tailed Student's *t*-test n = 4).

A defining feature of dPGA is the 3D geometry of dendritic particles, which may impact substrate characteristics important for neuronal adhesion and survival. Mammalian cells respond to nanometer-scale topological features (Kuo et al., 2014), however these features are beyond the resolution range of conventional light microscopy. We therefore utilized atomic force microscopy (AFM) to image the surface of glass coverslips coated with a 10 µg/ml solution of either PDL or dPGA. Substantial differences were detected in nanotopography. Imaging the PDL-coated glass revealed linear structures, suggesting strands of PDL, although much of the surface remains relatively flat. In contrast, the dPGA-coated surface exhibits pseudo-spherical structures, which may correspond to dendritic monomers that appear packed to cover essentially the entire surface. The dPGA coated surface also appears more complex, with greater variations in height, nanoscale ridges and valleys, compared to PDL (Figure 6A). This was confirmed by extracting the RMS roughness from the AFM scans, indicating a significantly greater RMS roughness of dPGA-coated glass coverslips than coverslips coated with PDL (average RMS roughness of 233 ± 33 for PDL vs. 497 ± 174 picometer for dPGA, *p* < .01, one-tailed Student's *t*-test, n = 4 section measured) (Figure 6B). These findings suggest that the increased nanotopological roughness associated with the dPGA-coated surface may contribute to its enhanced capacity to support cells in culture.

## Discussion

The synthetic polycationic peptides poly-lysine and poly-ornithine have been widely employed for decades as cell culture substrate coatings due to their reliability, reproducibility, and relative ease of use (Letourneau, 1975; McKeehan & Ham, 1976; Yavin & Yavin, 1974). These substrates may be augmented with a second layer of an ECM component, such as laminin or fibronectin, to enhance the capacity of the surface to support specific cell types (Clark et al., 1993; Hammarback et al., 1988). These modified surfaces have tremendous utility, however they are degraded by cellular proteolysis and it remains a challenge to maintain healthy cell cultures for extended periods of time, such as those required for the long-term differentiation of human iPSCs.

Here, we sought to identify a material that more optimally supports long-term cultures of neurons and other adherent cell types. We describe a novel substrate coating, dPGA, and show that it provides superior support for neural cells compared to standard polycationic peptides. We demonstrate that maintaining embryonic rat primary neocortical neurons on a dPGA coated glass surface resulted in higher neuronal densities in both short-term (7 days) and long-term (3 months) cultures compared to a standard PDL coated substrate. No differences were detected in the cellular morphology or electrophysiological properties of these neurons grown on dPGA compared to PDL. As a base layer under a laminin coating, we found that dPGA could replace the polycationic polypeptide PLO to more effectively support the growth and differentiation of human iPSC-derived cortical, hippocampal and dopaminergic neurons. Further, we documented a reduction in the number of non-neuronal cells and an increase in the number of neurons in cultures of iPSC-derived cortical and dopaminergic neurons grown on dPGA-laminin compared to a standard PLO-laminin coating. We show that the increased neuronal density found on a dPGA-coated substrate is not due to greater initial cell adhesion, but to an increase in cell survival that is established within the first 3–7 days of culture. Finally, examining the cell culture surfaces using AFM indicates that a dPGA coating provides enhanced nanoscale roughness compared to PDL.

### Higher Neuronal Density of Primary Cortical Neuron Cultures Grown on dPGA

A poly-lysine coating is the current substrate of choice in many protocols for primary neuronal cultures (Banker & Goslin, 1998). Here we detected a 50–96% increase in the number of surviving embryonic rat primary neocortical neurons after 7 weeks and an ∼300% increase after 3 months when cultured on dPGA compared to PDL. These findings indicate that dPGA is a superior coating for short-term and long-term support of this widely used primary neuronal culture.

A variety of substrate characteristics influence how cells interact with a surface, including surface charge (Ohgaki et al., 2001; Xu et al., 2009) and nanotopography (Spatz & Geiger, 2007; Vogel & Sheetz, 2006). The amine functional groups in PDL and dPGA are positively charged at physiological pH, and positively charged surfaces are well documented to promote neuronal adhesion and growth (Chen et al., 2018; McKeehan & Ham, 1976). While the branched core structure of dPGA is fundamentally different from PDL, the electrostatic surface charge of the two polymers is predicted to be similar. PDL and dPGA likely function in a similar fashion, acting as a bridge between the negatively charged glass substrate and negatively charged membrane phospholipids in the plasma membrane. Alternatively, polycationic polymers may engage cellular receptors that mediate adhesion, however a receptor for poly-lysine or other polycations has not been identified. In addition to this bridging mechanism, substrate-bound polymer may capture and present *bona fide* ECM macromolecules secreted by the seeded cells or adsorbed from serum in the culture medium.

Imaging nano-topography using AFM revealed that coating a clean glass coverslip with dPGA produces a surface with greater nanoscale roughness compared to coating with PDL. This suggests that the relatively “rough” 3-dimensional architecture of a dPGA coating may contributes to the improved support for neuronal cultures. Compared to coating with a linear polymer such as PDL, the adsorbed dPGA may increase the available surface area for binding and thereby provide a synthetic extracellular substrate matrix that better supports cell growth, survival and differentiation.

We detected a substantial increase in the number of glial cells in the primary neocortical cultures between 7 days and the 3 months *in vitro*. These likely originated from the expansion of the limited population of non-neuronal cells present at 7 DIV over the following 7 weeks. Astrocytes are an integral part of neuronal tissue and factors secreted by the astrocytes *in vitro* likely contributed to maintaining neuronal health over the 3-month period (Banker, 1980; Jones et al., 2012; Kaech & Banker, 2006).

While the coatings initially used for neuronal cultures were either proteins or polypeptides, other synthetic/non-peptide cationic linear polymers have been investigated for their capacity to support neuronal adhesion and growth (Ji et al., 2019; Lakard et al., 2005; Landry et al., 2018; Schmidt et al., 1997; Vancha et al., 2004). dPGA belongs to a class of purely synthetic highly branched polymers. Notably, the complete absence of peptide bonds in the dPGA backbone renders it highly resistant to cellular proteases and this is predicted to improve substrate stability and enhance the long-term maintenance of cells in culture. Our findings demonstrate that synthetic extracellular matrices constructed from macromolecules that diverge from linear polypeptide polymer structures can provide enhanced support for long-term cell culture.

### dPGA Supports iPSC-Derived Neurons

In cultures of iPSC-derived cortical and dopaminergic neurons grown on dPGA-laminin, we detected a reduction in the number of non-neuronal cells and an increase in the number of neurons, compared to cultures on PLO-laminin. We speculate that this is due to early events after seeding that favored the differentiation of neural progenitor cells into the intended neuronal type on a dPGA coating. Early differentiation into post-mitotic cells, such as cortical or dopaminergic neurons, would result in fewer actively dividing neural progenitor cells, thereby resulting in reduced numbers of glia, non-dopaminergic neurons, and other neural progenitor cells.

Unlike the iPSC-derived cortical and dopaminergic neurons, we found that the combination of dPGA and laminin supported the survival, growth and differentiation of hiPSC-derived hippocampal neurons similar to a standard PLO-laminin coating. This may reflect differences in the differentiation protocols used for each cell type. Cortical and dopaminergic protocols yielded a relatively low percentage of neurons (10% to 35% yield) compared to the protocol used to differentiate hippocampal neurons (55% to 65% yield). As such, the conditions to differentiate the hippocampal neurons may be approaching a maximum yield, that was not enhanced by the dPGA substrate.

We did, however, note reduced cell aggregation in hiPSC-derived dopaminergic and hippocampal neuronal cultures when grown on a dPGA-laminin combination compared to the standard PLO-laminin cultures, suggesting improved stability of the polyglycerol-based polymer compared to PLO. The aggregation of cells into clumps in culture, such as during the formation of organoids or neurospheres, is an indication of preferential cell-cell adhesion and relatively poor adhesion to the underlying substrate (Xie et al., 2017). In long-term cultures, cell aggregation often occurs in response to the degeneration of a substrate that was previously adhesive. In such a case the cells aggregate with each other rather than adhere to the degraded non-permissive substrate. Large aggregates of hiPSC-derived neurons found in long-term cultures complicate cell counting, analyses of single cell-morphology, isolation of single cells for transcriptomic analysis and electrophysiological recording. Ultimately the weak adhesion between the cells and the degraded substrate becomes insufficient to tether the aggregates and the cultures become non-viable. Our findings indicate that the enhanced stability of a dPGA substrate significantly reduces cell aggregation during long-term cell culture.

### dPGA-Coated Substrate Does not Detectably alter Neuronal Physiology

A concern when employing a novel substrate is that it might adversely or differentially affect cellular physiology or function. Critically, we did not identify any differences in the expression of functional markers expressed by the primary cortical neurons maintained on either PDL or dPGA. Neurons grown on dPGA-coated surfaces formed functional synaptic connections with neighboring neurons and had electrophysiological properties indistinguishable from neurons grown on PDL-coated surfaces.

## Conclusion

dPGA is relatively inexpensive to synthesize, easy to use and has a long shelf-life. Assessment of cultures of rat primary neocortical neurons and several types of human iPSC derived neurons indicate that dPGA can be readily substituted for PLL, PDL, or PLO-based coatings and is an improved substrate coating for enhanced support of long-term adherent cell culture.
